# Incorporating Real‐World Variability in Clinical IBD Research

**DOI:** 10.1111/jep.70117

**Published:** 2025-05-06

**Authors:** Anje A. te Velde

**Affiliations:** ^1^ Tytgat Institute for Liver and Intestinal Research, Amsterdam UMC location AMC Amsterdam Gastroenterology Endocrinology Metabolism (AGEM) University of Amsterdam Amsterdam The Netherlands

1

Every problem is embedded in a greater whole and should not be oversimplified by selectively focusing on only the variables that are easily identified and measured.

This statement highlights the interconnectedness and complexity of problems within larger systems. It suggests that problems cannot be fully understood or addressed by focusing solely on the variables that are most apparent or easiest to identify. To truly grasp a problem, one must consider it within the context of the broader system or environment in which it exists, acknowledging all relevant factors—even those that may be harder to observe or measure.

In other words, simplifying a problem by overlooking less obvious variables can lead to incomplete or inaccurate solutions. This perspective aligns with systems thinking, which emphasizes the importance of considering the entire system, recognizing the interplay between various elements, and understanding how changes to one part can affect the whole.

In the article by Sturmberg and Mercuri, 2024 [1] Peter Drucker is quoted pointing out

An important distinction between ‘doing things right’ and ‘doing the right thing’, which recognized that all problems are embedded in a context and thus can only be understood within their unique contextual setting. Contemporary research practices in clinical medicine often regards contextual factors as potential confounders that will bias effect estimates and thus must be avoided. However rigorous, research devoid of context ultimately deprives users of understanding of the support factors that make research transferable to policy decisions or managing care of individual patients—it stands in the way of ‘doing the right thing’ in ‘real life’ settings.

For my own understanding, I needed to search the internet for further clarity. I came across explanations from several sources that distinguish between ‘doing things right’ and ‘doing the right things’. ‘Doing things right’ refers to following rules, policies, procedures, and norms, while ‘doing the right things’ involves aligning with our values and moral compass in pursuit of something greater. This immediately reminded me of a topic I discussed in a previous paper on current practices in clinical research related to inflammatory bowel diseases (IBD) [2].

Therefore, I would like to expand on the broad impacts of environmental context on patient care and health outcomes for the conditions analyzed in this paper—diabetes, coronary heart disease, and cancer—by including thoughts on IBD, which I consider a prototype of complex disease. Due to its anatomical localization and our current understanding of immune reactions, this intestinal inflammation can be likened to a fire, manifesting as an enormous, uncontrolled adjuvant reaction. To extinguish this fire, it is essential to address the disease's complexity by simultaneously targeting all modifiable aspects: innate immunity, cytokines, microbiota, adaptive immunity cells, cytokines, and factors related to the (micro)environment [2].

The latter is particularly important, as IBD can also be considered a Western disease, with lifestyle factors playing a central role in its initiation. One of the main environmental factors associated with the onset and progression of IBD is the patient's diet [3, 4]. While the exact causative role remains unclear, diet is a key factor in shaping the composition and function of the microbiota, which in turn directly influences immune function [5, 6]. The Western pro‐inflammatory diet, which is high in fat, sugar, salt, and additives such as emulsifiers, while low in fiber (vegetables and fruits), contributes to the development of IBD [7, 8]. This dietary preference reduces the diversity of the gut microbiome and causes shifts in the relative abundance of certain key commensal taxa [9]. It is important to recognize that the status of the gut microbiome influences several vital aspects of clinical studies. For example, studies in cancer patients have shown that the response to checkpoint inhibitors (anti‐cancer biologics) varies depending on the type of microbiota present. Another consideration is that short‐term intervention studies with high‐fiber diets have demonstrated that rapid shifts in gut microbiota and metabolites are achievable [10].

Thus, diet can drive variations in the microbiota, adding to the complexity of the disease. However, most studies on microbiota composition do not account for dietary intake [11].

Moreover, although not all patients believe their diet is the cause of their IBD, more than 80% of IBD patients change their diet after diagnosis, and most report an improvement in symptoms [12].

Alongside this, many patients seek ways to improve their quality of life, such as making dietary adjustments [13, 14]. Given these observations, it is crucial to consider both dietary and microbiota status when conducting clinical studies [15].

However, patients often do not report the use of complementary treatments or lifestyle changes, meaning that physicians conducting clinical research may remain unaware of these factors unless explicitly addressed in the research protocol. In most research protocols, outcome measures and metadata focus primarily on clinical parameters, and although a variety of patient‐reported outcome measures have been developed, diet and lifestyle are generally not included [16].

Most studies begin by acknowledging that the etiology of IBD is not fully understood, but it is believed to result from a complex interplay between a dysregulated immune response, the presence or absence of specific gut microbiota, and environmental factors—including diet and other lifestyle factors—along with genetic susceptibility.

Given the strong link between lifestyle and IBD, I wondered whether lifestyle factors are considered in the design of randomized controlled trials (RCTs), which are considered the gold standard for proving the efficacy of newly developed biologics in treating IBD. This consideration is particularly important, as studies have shown that microbiota and their endogenous metabolites can serve as predictive tools for assessing treatment responses to various biologics. For example, it was found that in patients with a dysbiotic microbiota, vedolizumab appeared less effective [17]. Additionally, a stool‐based model was developed to predict that over 50% of nonresponders would fail to respond to any anti‐inflammatory intervention. The Bact2 inflammatory enterotype, which is considered dysbiotic, was likely responsible for this finding and has been linked to systemic inflammation [18].

To determine whether lifestyle factors are considered in clinical IBD studies investigating newly developed medications, a cross‐section of recent RCTs, systematic reviews (SR), and meta‐analyses (MA) on IBD was reviewed, with a focus on mentions of lifestyle. In most cases, except for a few studies that include smoking behavior, individual lifestyle factors that might influence trial outcomes or change during the course of the study are not addressed, see Table 1. Since patients with IBD often manage various lifestyle factors, neglecting to consider their potential role during the study leads to significant flaws in the research.

**Table 1:** Summary of recent intervention studies in IBD, highlighting whether lifestyle or dietary factors were addressed.

Year	Intervention or RCT analysis	Type of study	Lifestyle, diet mentioned	Reference
Ma et al. [2018]	Definitions of response and remission	RCT	Not	[19]
Ma et al. [2018]	Outcome set definition	SR	Not	[20]
Melmed et al. [2018]	Concomitant Immunomodulator and Biologic Therapy	RCT	Not	[21]
Johnson et al. [2020]	Trial participation	RCT	Not	[22]
Davies et al. [2020]	Tofacitinib	RCT	Not	[23]
Alipour et al. [2021]	Deep remission with anti TNF	RCT and RWE	Not	[24]
Macaluso et al. [2021]	Head to head comparison	RCT and RWE	Not	[25]
Lee et al. [2021]	Smoking and anti‐TNF	SR and MA	Smoking	[26]
Magro et al. [2023]	Time trends in biological therapies	SR and MA	Strategy considered: combining drugs and nutrition	[27]
Hu et al. [2023]	Vedolizumab	SR and MA	Not	[28]
Gao et al. [2023]	IL‐23 and IL‐12 inhibitors	MA	Not	[29]
Gordon et al. [2023]	Infliximab	SR	Smoking	[30]
Hu et al. [2024]	Biosimilar anti TNF	SR and MA	Not	[31]
Zheng et al. [2024]	Upadacitinib	SR and MA	Not	[32]
Vieujean et al. [2024]	Study recruitment only	RCT	Smoking	[33]
Gordon et al. [2024]	Reporting risk of bias	RCT	Not	[34]
Leão Moreira et al. [2024]	Multiple component outcome	RCT	Smoking	[35]
Roblin et al. [2024]	Risankizumab	RCT	Smoking	[36]
Ferrante et al. [2024]	Mirikizumab	RCT	Not	[37]
D'Haens et al. [2024]	Vedolizumab to prevent postoperative recurrence	RCT	Smoking	[38]
Peyrin‐Biroulet et al. [2024]	Risankizumab and Ustekinumab	RCT	Not	[39]
Sands et al. [2024]	Ozanimod	RCT	Not	[40]

Abbreviations: MA, meta‐analysis; RCT, randomized controlled trial; RWE, real‐world evidence; SR, systematic review.

One recent meta‐analysis [27] demonstrated that, over the past decades, the clinical outcomes of patients receiving biological therapies have remained stable. This stability may be due to a therapeutic ceiling effect. The authors note that, over time, the design of clinical trials has been adapted to address heterogeneity. However, they also conclude that only limited baseline characteristics were available, which resulted in a lack of options for performing sub‐analyses and identifying patient subpopulations.

Heterogeneity in IBD is well acknowledged, with two main subtypes—Crohn's disease (CD) and ulcerative colitis (UC)—and a third, smaller group of indeterminate or IBD‐unclassified cases, comprising around 7% of the patient population. It is likely that additional disease subtypes can be distinguished in the clinic, for example, based on the natural history or location of the disease [41]. Genetic studies conducted since 2008, including large genome‐wide association studies (GWAS), have provided valuable insights into IBD susceptibility genes and the pathways that regulate mucosal immunity, highlighting the disease's heterogeneity and complexity [42]. However, these studies did not identify any distinct subtypes. More recently, epigenetic studies have revealed subpopulations of IBD patients that exhibit different responses to biological treatments [43]. In the context of other autoimmune diseases, researchers have identified two subtypes: one characterized by high metabolism and the other by high inflammation, each with distinct molecular profiles [44].

Finally, several studies are comparing the two main IBD subtypes using various omics techniques, and this multi‐omics approach will eventually create an IBD interactome [45]. Further subgrouping could involve investigating whether separate interactome networks can be distinguished in CD and UC, defined by molecularly homogeneous pathological mechanisms. It has been suggested that these distinct subgroups could be treated with specific medications, yet there is still little consideration of possible lifestyle interventions for this complex condition [46].

Taken together, recent studies and advancements in computational models that integrate increasing amounts of data are helping to identify different IBD subtypes beyond the CD and UC division. This progress may prove valuable in unraveling the disease's variability and complexity, including its interaction with the environment and lifestyle. Ultimately, embracing this broader context in the design and execution of clinical trials will be crucial.

## 2 Setting a Step Forward

**Figure 1:**
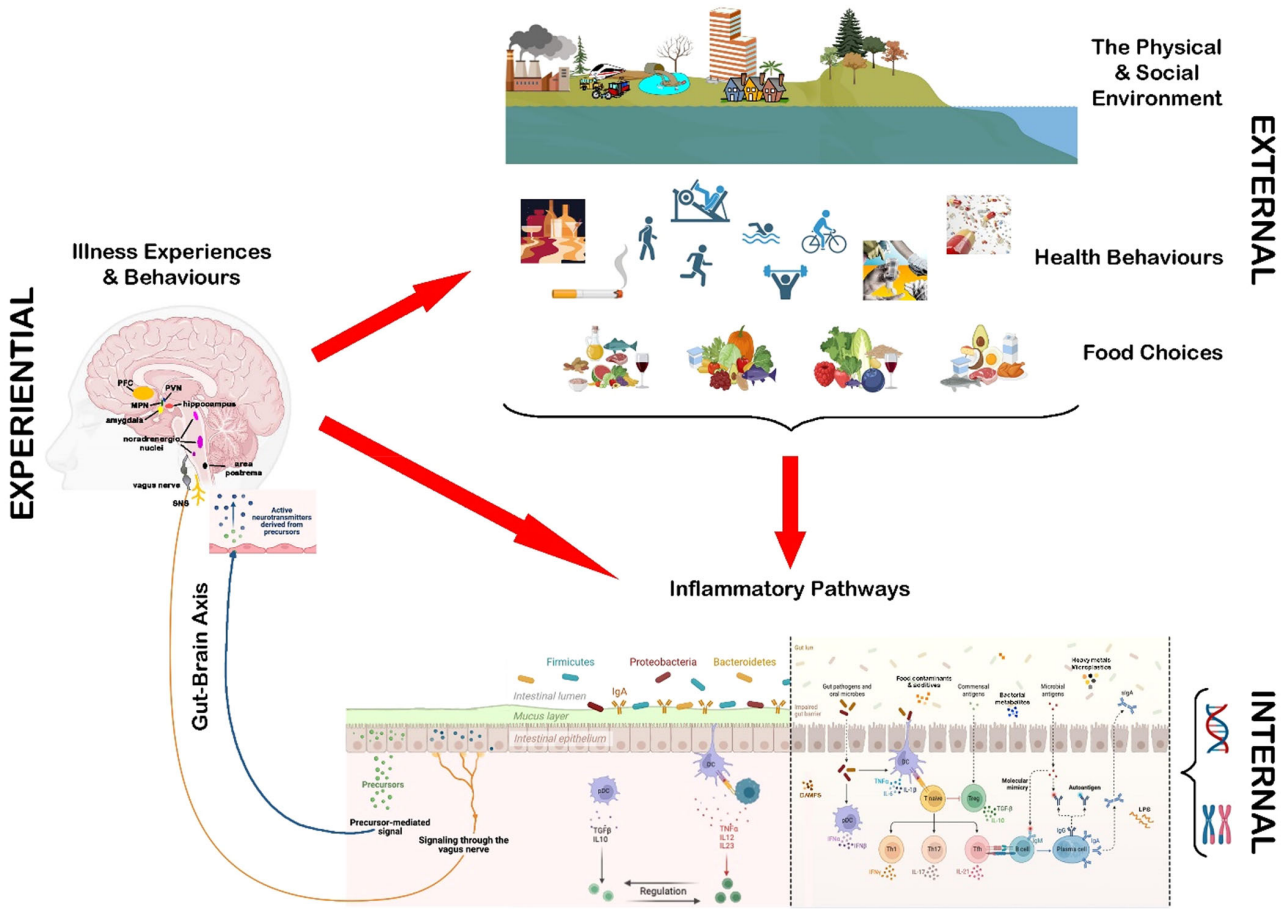
The relationship between experiential illness experiences and behaviors on external and internal environment. Figure is credited to Prof. Dr. J. Sturmberg.

In their article, Sturmberg and Mercuri [1] propose a mental framework for addressing the challenges of understanding interconnected problems in disease, marking a small step forward. To effectively study real‐world variations, a different approach is needed—one that embraces pragmatic trials. This methodology offers a way to identify patterns that explain the heterogeneity in treatment effects. Ideally, through such trials, biomarkers and distinct patient phenotypes can be identified, leading to a deeper understanding of treatment response variability, see (Figure 1).

How is the implementation of pragmatic trials in IBD evolving, and is lifestyle considered a factor contributing to heterogeneity to better reflect real‐world conditions? In recent years, pragmatic clinical trials have gained attention, becoming one of the five key focus areas in IBD research for 2024 [47]. One study on pragmatic clinical research highlights several priorities: achieving optimal outcomes for all patients, integrating biomarkers, and optimizing treatment sequences. The researchers emphasize the need to explore in comparative effectiveness studies not only pharmacological agents but also dietary and microbiome‐directed interventions as adjuncts to standard therapy. Additionally, they refer to existing literature that suggests that various lifestyle factors, beyond diet, may contribute to IBD flare risk [48]. Although there is growing support for dietary interventions as supplementary treatments for IBD, the role of environmental factors and lifestyle changes—including diet—as potential confounders in clinical trials has not been considered in this pragmatic clinical trials. This oversight may limit the applicability of trial results to real‐world practice. Another study established a consensus on designing pragmatic clinical trials to assess treatment effectiveness in real‐life settings, outlining 25 key statements for optimal trial design [49]. While there is an intention to incorporate real‐world variability into these studies, environmental and lifestyle factors are again overlooked. This gap is further reflected in the core outcome set for real‐world IBD data, developed by an international multidisciplinary working group, which did not explicitly address these factors, despite including dietitians in the discussion [50].

For pragmatic trials and truly representative real‐world studies in IBD, it is essential to incorporate all measurable environmental factors that influence disease progression. The necessary tools—such as food frequency questionnaires, microbiome analyses, and studies on environmental triggers—are available and should be utilized [51, 52, 53].

## Conflicts of Interest

The author declares no conflicts of interest.

## Data Availability

The author has nothing to report.
